# Anogenital distance in the etiology of chronic prostatitis: does it lead to novel surgical treatments?

**DOI:** 10.3389/fruro.2024.1418007

**Published:** 2025-01-07

**Authors:** Ayhan Verit, Fatma Ferda Verit

**Affiliations:** ^1^ Department of Urology, University of Health Sciences, Fatih Sultan Mehmet Hospital, Istanbul, Türkiye; ^2^ Department of Gynecologic Oncology, Istanbul Cerrahpaşa University, Istanbul, Türkiye

**Keywords:** Chronic prostatitis (CP), chronic pelvic pain syndromes (CPPS), anogenital distance (AGD), etiology of CP, treatment of CP

## Introduction

Chronic prostatitis (CP), among other chronic pelvic pain syndromes (CPPSs) with largely unknown etiologies, is a pathology that results in a significantly decreased quality of life in addition to a significant economic burden (1, 2). The worldwide prevalence of CP was reported to range between 2% and 25% but overall is thought to be near the upper limit (3, 4). CP/CPPSs are a group of conditions that are thought to be related to the prostate gland in men that continues or worsens over a long period of time, usually at least more than 3 months. The main symptoms are body aches, lower back and genital area pain, dysuria, and problems with emptying one’s bladder (1–4). Unfortunately, there is neither a widely confirmed classical treatment option nor a remedy for curing the disease other than relieving the symptoms of CP. The combination of a malfunctioning immunity system and increased oxidative stress induced by local ischemia is among the reported pathophysiological mechanisms in the etiology of CP alongside the ranking endocrine/neurological/infectious ones (5–8).

Vinclozolin is an antiandrogenic drug that blocks the activity of gonadal hormones in male reproductive organs, including the perineum (9). Vinclozolin exposure in the embryological stage has been shown to reduce anogenital distance (AGD), which is simply the length of the skin between the center of the anus and the junction between the smooth perineal skin and skin of the scrotum or penis and is represented as AGD^AS^ or AGD^AP^ respectively (9–11). There are some clinical studies that show that AGD may be associated with well-known androgen-sensitive pathologies such as prostate cancer (PC), male infertility, hypospadias, cryptorchidism, and even benign prostatic hyperplasia (BPH) and premature ejaculation (11–20). Thus, this physical measurement has been suggested as a diagnostic helper in clinical evaluations. However, unlike the current literature, in this text, our aim is to indicate the prospect of using this physical marker to define the etiology of CP, rather than an endocrine-related disorder, by measuring the physical compression inside the narrowed perineum. Additionally, in this article, we would like to improve our pioneering hypothesis on this topic with additional evidence and a discussion on possible surgical treatments (21).

## The main text

**Figure 1:**
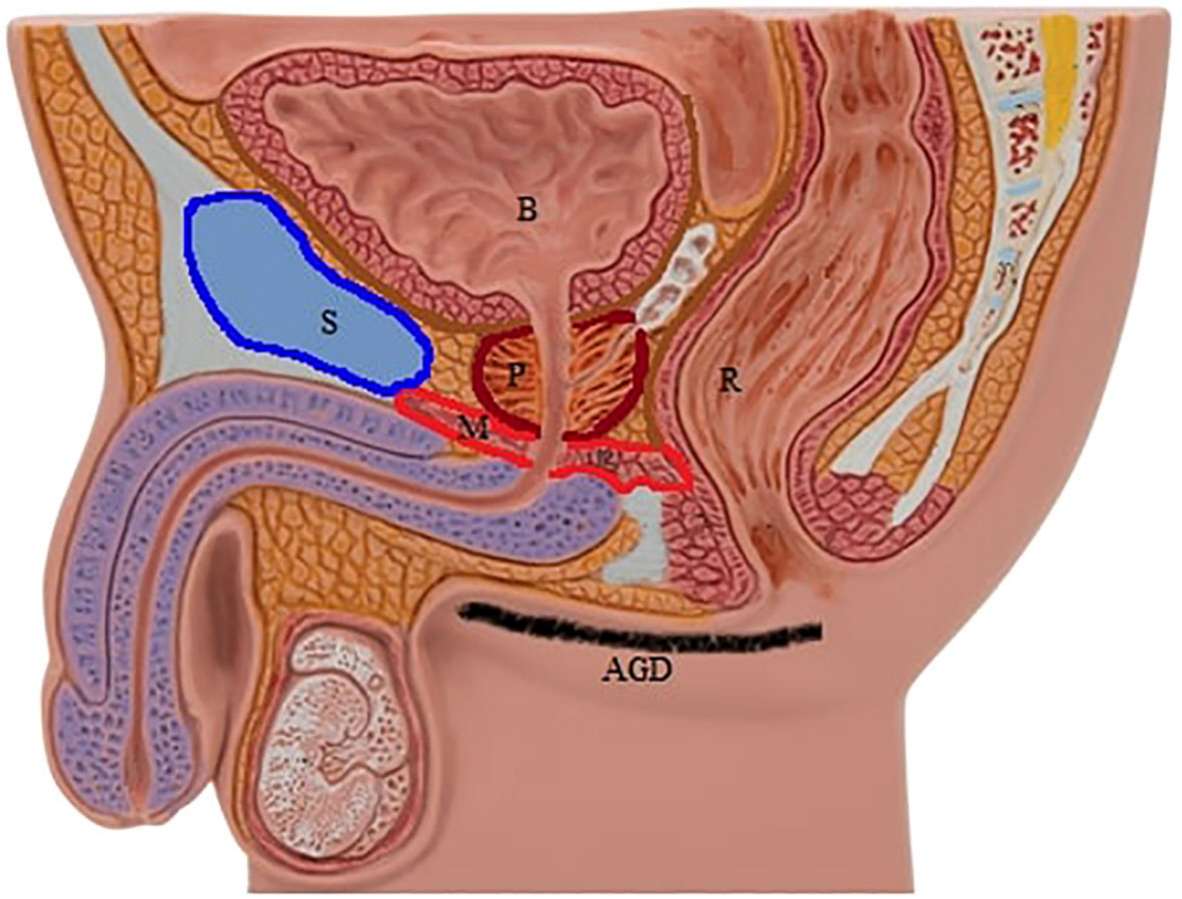
Note the trapped prostate and surrounding structures in relation to the anogenital distance. P, prostate; B, bladder; R, rectum; S, pubic symphysis; M, pelvic floor muscles; AGD^AS^, anogenital distance (anus-scrotum).

The perineal structures are the bottom floor of the pelvis and can be considered geometrically as the apex of a reversed cone. Whereas the superficial anatomical tip of this cone is in an anterior-posterior line, namely the AGD, the internal side of this line extends between the lower urinary tract, mainly the urethra and rectum, which is exactly the location of the prostate, a unique parenchymal organ without a true capsule in this zone (**Figure 1**). Thus, we hypothesize that a narrowing of this internal side of the cone may directly and constantly compress prostatic tissue with its associated neurovascular pedicle and result in chronic hypoxia (21). We believe that the *Denonvilliers’* fascia in the anterior side of the rectum, as a very tight structure, has a special role in reflecting the pressure on the prostate more than the rectum itself in this physiopathological process. The main factor that restricts the prostate on the anterior side is the os pubis. Moreover, the stabilizing ability of the urethra that extends inside the prostate, and also the prostatic ligament and endopelvic fascia, are among the apparatuses to make the prostate immobile and exert chronic pressure on prostatic tissue (**Figure 1**). As a result, the prostate is trapped between internal perineal structures, mainly between the os pubis and rectal fascia in antero-postero direction, and the strength of this pressure can be quite variable on an individual basis due to the length of a defined area, namely the AGD. Furthermore, uro-oncologic surgeons have experienced difficulty during the dissection of the prostate from the surrounding tissues in radical prostatectomy which we think is a clear example of a trapped prostate at the bottom of the pelvis. Moreover, eliminating the constipation problem in patients with CP is the initial goal of management strategies that aim to discharge internal rectal pressure. It is difficult to confirm all these physical dynamic pressure disseminations *in vivo*. Overall, while the internal side of the AGD begins anteriorly on the internal face of the os pubis, including the urethra, endopelvic facia, and prostatic ligament that fix the prostate in its longitudinal axis and also restrict its lateral movements, it ends posteriorly at the rectum and mainly its *Denonvilliers*’ fascia. Nevertheless, a narrowed perineum is supposed to result in two parallel blocks of compression on the prostate and its accessories, such as the seminal vesicles and vascular structures that lie posterolaterally of the prostate. The severity of the pressure mechanism may increase by shrinking the above-mentioned area which can be estimated by measurement of the AGD (**Figure 1**). Although the AGD is only a one-dimensional marker, we think that this simple measurement can also provide a good estimation of the size of the tip of the cone in two and three dimensions. It is noteworthy that this zone is also the point of the abdominal cavity that incurs the highest pressure due to gravity, especially in a standing position. While this significant pressure gives rise to “pelvic organ prolapsus” in women, there is not a similar pathology in men that supports our hypothesis about trapped strain at the region. Furthermore, this standing-up position specific to the human species among land mammals, may increase the severity of lower abdomen venous pathologies that are all thought to have a common origin (22). The most common one is rectal hemorrhoids and others include pelvic venous diseases, namely “pelvic congestion syndrome” associated with CPPS in women and a varicocele in men (23, 24). Flavonoids, with their healing efficacy for vessels, are the current systemic medication for rectal hemorrhoids and are also reported to be effective in CP treatment which confirms varicose impairment extends to the prostate in theory (25, 26). Thus, increased hydraulic venous pressure seems to lead to intra-prostatic strain, or vice versa. The physical pressure on the prostate leads to intra and/or periprostatic varicose disorders, which appear clinically as CP. In summary, as a sub-hypothesis, we argue that the prostate is also the target of varicose disturbances, as is its anatomical neighborhood, as indicated by the literature on the treatment options for CP.

Moreover, it can be claimed that this external pelvic pressure on the prostate can be transmitted to the urethra inside the prostate which may be another physical factor that decreases urinary flow rate. Currently, this can be even calculated by low-cost digital home uroflowmetry systems which are appropriate for telemedicine (27). At present, prostate-relaxing surgical procedures may also have the potential to reduce bladder outlet obstruction (BOO). However, BOO due to the CP was excluded from this discussion. Furthermore, some improvements in bacterial prostatitis (5%-10%) were also reported such as the prescription of nutraceutical antioxidant products alongside appropriate spectrum antibiotics (28). This limited group was also excluded from this discussion due to its well-known standard medical treatment approach.

We hypothesize that the histological appearance of the aforementioned mechanism, i.e., a local inflammatory reaction related to a maladaptive immune response and oxidative stress due to chronic hypoxia, is noted as CP in prostate biopsy reports when searching for PC. It is noteworthy that the definition of histological CP could be found in a high rate of biopsy materials, with 60%-80%, that were indicated for suspicion of PC with or without symptomatic prostatitis and no bacterial induction was confirmed in most of them (29, 30).

Although the hypothesis of endocrine disturbance in the etiology of CP was excluded in the present discussion due to the aim of this study, we should mention that *in-utero* antiandrogens/estrogens such as vincozolin/dinesterol were shown to induce dose-dependent histological post-pubertal prostatitis in animal models (9, 31). It may be claimed that initial androgen deficiency, which results in a shortened AGD, is also the histological and/or clinical basis for CP, enhancing the impact of ischemia-induced CP later in adulthood life described in the present text. Nevertheless, in another explanation, we speculate that there is no endocrinological etiology in CP without the mechanism of a shortened AGD and the present mechanism begins in the perinatal period and progresses throughout life via accumulation of the ischemic inflammation.

In relation to the neurological etiology that was also excluded from the discussion, we would like to remind the reader that perineal “pain” and “tenderness” are some of the common symptoms of CP that may be caused by the prostate itself or the perineum which are the direct targets of the aforementioned synchronous physical forces and chronic ischemia on the external side of the prostate (32). It should also be considered that the afferent pathways of the prostate related to pain transmission are complicated and not fully understood (33).

Overall, it should be strongly emphasized that CP, and in general CPPSs, are a group of not fully understood clinical disorders without confirmed pathophysiology, and in addition, they are difficult to investigate with well-designed study protocols. However, in this study, we tried to introduce a unique perspective on this clinical status via a discussion of the pure physical forces supported by the literature that can be predicted by simple measurements at a routine physical investigation besides the possible classically reported etiologies.

For clinical practice, we think that AGD is a simple measurement that can be applied during the inspection phase of a digital rectal investigation for CP that can help the physician confirm the diagnosis of CP. Moreover, if our hypothesis is confirmed by further well-designed experimental and clinical studies, some novel surgical techniques, such as ureterolysis in retroperitoneal fibrosis or for some gynecologic operations, to loosen the prostate from the fascia-like connective surrounding structures to mitigate the detrimental effects of chronic tension may possible surgical treatments for CP, especially for the selected cases who have a reduced AGD. Regarding the aim of the study, we suggest the release of the pubo-prostatic ligaments, and endopelvic, levator, and prostatic facias to possibly help to reduce pressure on the prostate in patients with CP but care must be taken not to destroy the neurovascular bundle to preserve erection capability. All these structures and their dissection were defined in detail by Walsh (34) Current surgical interventions include transurethral removal of prostatic tissue (TURP), radical TURP, transurethral vaporization of the prostate (TUVP), and even radical prostatectomy for the refractory cases, however, the efficacy and safety of these surgical treatment options are not sufficient for the evidence-based practice and are generally conducted for chronic bacterial prostatitis (4). Nevertheless, the present defined prostate-releasing surgeries, especially minimally invasive laparoscopic surgery with/without robot assistance, cannot be considered unsafe in comparison with those that target the removal of prostatic tissue. However, as an interesting noteworthy critique, novel surgical methods for the prostate are supposed to have an upward trend in cost and result in a significant load on the healthcare system (35).

To conclude, apart from the current knowledge, AGD can not only provide information about the male urogenital system pathologies via embryologic hormonal pathways, but we also hypothesize that AGD may be a physical sign of histological/clinical prostatitis due to a shortened perineum.
